# Food Consumption as a Modifier of the Association between *LEPR* Gene Variants and Excess Body Weight in Children and Adolescents: A Study of the SCAALA Cohort

**DOI:** 10.3390/nu10081117

**Published:** 2018-08-18

**Authors:** Aline dos Santos Rocha, Rita de Cássia Ribeiro-Silva, Gustavo Nunes de Oliveira Costa, Camila Alexandrina Figueiredo, Laura Cunha Rodrigues, Sheila Maria Alvim Matos, Rosemeire Leovigildo Fiaccone, Pablo Rafael Oliveira, Nadya Helena Alves-Santos, Ronald E. Blanton, Maurício Lima Barreto

**Affiliations:** 1 Departamento Ciência da Nutrição, Escola de Nutrição, Universidade Federal da Bahia, Av. Araújo Pinho, 32, Canela, CEP: 40.110-150, Salvador, BA Brasil; ritaribeiroufba@gmail.com; 2 Instituto Gonçalo Moniz (IGM), Fundação Osvaldo Cruz—FIOCRUZ-Bahia, Av. Waldemar Falcão, 121, Candeal-Salvador/BA, CEP: 40296-710, Salvador, BA, Brasil; gustavokosta@gmail.com (G.N.d.O.C.); pablorafael_ssa@hotmail.com (P.R.O.); mauricio@ufba.br (M.L.B.); 3 UNIFACS—Universidade Salvador, Laureate International Universities, Rua Doutor José Peroba, 251, Edf. Civil Empresarial, Sobreloja, STIEP, CEP: 41770-235, Salvador, BA, Brasil; 4 Departamento de Ciências da Biorregulação, Instituto de Ciências da Saúde, Universidade Federal da Bahia, Av. Reitor Miguel Calmon. s/n, Vale do Canela, CEP: 40110-100, Salvador, BA, Brasil; cavfigueiredo@gmail.com; 5 Department of Epidemiology and Population Health, London School of Hygiene and Tropical Medicine (LSHTM), Keppel Street, London, WC1E 7HT UK; laura.rodrigues@lshtm.ac.uk; 6 Instituto de Saúde Coletiva, Universidade Federal da Bahia, Rua Basílio da Gama, s/n, Canela, CEP:40.110-040, Salvador, BA, Brasil; sheilaalvim@gmail.com (S.M.A.M.); rose.fiaccone@gmail.com (R.L.F.); 7 Instituto de Matemática, Universidade Federal da Bahia-Av. Adhemar de Barros, s/n-Ondina, CEP: 40.170-110, Salvador, BA, Brasil; 8 Cidacs—Centro de Integração de Dados e Conhecimentos para Saúde, Instituo Gonçalo Muniz, Fundação Owaldo Cruz, Ministério da Saúde, Parque Tecnológico da Bahia, Rua Mundo, 121, Trobogy, CEP: 41745-715, Salvador, BA, Brasil; 9 Observatório de Epidemiologia Nutricional, Departamento de Nutrição Aplicada e Social, Instituto de Nutrição Josué de Castro, Universidade Federal do Rio de Janeiro-UERJ. CEP: 21941-590, Rio de Janeiro, RJ, Brasil; nadyasaint@hotmail.com; 10 Center for Global Health & Diseases, Biomedical Research Building (BRB), Room 425, Case Western Reserve University, 2109 Adelbert Rd, Cleveland, OH 44106, USA; reb6@case.edu

**Keywords:** overweight, obesity, leptin receptor gene, children and adolescents, food consumption

## Abstract

No studies showing that food consumption is a modifier of the association of variants of the leptin receptor gene (*LEPR*) with body weight have involved a Brazilian population. The aim of this study was to evaluate the modifying effect of dietary intake on the association between the *LEPR* gene and excess weight. In this study, 1211 children and adolescents aged 4–11 years were assessed. Participants were genotyped for 112 single-nucleotide variants of the *LEPR* gene. Anthropometric measurements were performed, and dietary data were obtained. Logistic regressions were used to study the associations of interest. Of the participants, 13.4% were overweight/obese. The risk allele (G) of the rs1137100 variant was associated with excess weight in individuals with fat consumption below the median (odds ratio OR = 1.92; 95% confidence interval CI = 1.18–3.14), with daily frequency of consumption of drink/artificial juice (OR = 2.15; 95% CI = 1.26–3.68) and refined cereals (OR = 2.17; 95% CI = 1.31–3.62) above the median. The risk allele (G) of variant rs1177681 was also associated with excess weight (OR = 2.74; 95% CI = 1.65–4.57) in subjects with a daily frequency of refined cereal consumption above the median. The association between *LEPR* and excess weight can be modulated by the type and distribution of dietary fatty acids, sugary drinks, and refined cereals.

## 1. Introduction

Excess weight in childhood and adolescence is a growing trend and it constitutes one of the major causes of morbidity in these life stages A population-based survey conducted in Brazil estimated that 47.8% of children aged 5–9 years and 25.4% of adolescents aged 10–19 years were overweight/obese [1]. At present, there is growing interest in understanding complex diseases, such as overweightness/obesity, which are characterized by a multiplicity of interactions between genetic and postnatal exposures of various types (social, economic, cultural, psychosocial, and environmental) that maximize the effects of genetic polymorphisms on excess weight gain [2,3]. Changes in dietary intake, such as increased consumption of ultra-processed foods (characterized by high energy density and foods rich in saturated fat and simple carbohydrates) combined with reduced physical activity levels and an increasingly sedentary lifestyle are important contributors to the current obesity issue, and related chronic diseases associated with excess weight gain [4].

Genome-wide association studies (GWAS), have demonstrated numerous genetic susceptibility loci associated with the risk of obesity in adults, many loci identified in adults also play a role in the pediatric setting. [5]. Of the genes associated with overweight and obesity, those related to appetite control through the hypothalamus deserve special mention. Single nucleotide variants (SNVs) in the leptin receptor gene (*LEPR*) and other genes in the leptin-melanocortin hypothalamic pathway (*LEP*, *POMC-ADCY3*, *PCSK1*, *MC4R*, *BDNF*) have been reported in humans with obesity-related traits, and in children specifically [6]. Genetic variations in *LEPR* were already reported to be associated with a reduced capacity of leptin to regulate body weight and energy homeostasis, a process known as leptin resistance, which can lead to obesity-related phenotypes [7].

Several studies have demonstrated the association between the *LEPR* gene, SNVs, and overweightness/obesity in children and adolescents [8,9,10], but other results are discordant [11,12]. In fact, gene-environment interactions may explain the heterogeneity of the results [13]. Studies have reported the effects of nutrients, such as saturated fats, modulating the associations between these genetic variants, and overweightness and obesity to potentiate the obesogenic effect of the *LEPR* on the expression of genes linked to overweight/obesity [14,15]. However, few studies have investigated the mechanisms the interactions between food components and *LEPR* gene variants with regard to effects on excess weight. Moreover, none of them have involved Latin American populations with a high level of miscegenation, as is seen in the northeastern Brazilian population.

Considering the limited results and the relevance of this topic for the promotion of health, the aim of this study was to evaluate the modifying effect of diet on the association between the *LEPR* gene, and excess weight in children and adolescents. We hypothesized that food components can modulate/modify the expression of *LEPR* gene variants on the overweight/obese phenotype. A better understanding of these interactions has the potential to support overweightness and obesity prevention through dietary recommendations.

## 2. Materials and Methods

### 2.1. Study Design and Population

This was a cross-sectional population-based study nested in a cohort study of asthma risk factors in Salvador, titled the Social Changes, Asthma and Allergy in Latin America (SCAALA) [16]. The sample consisted of 1445 children and adolescents (4–11 years of age) randomly selected from 20,000 families, and covering a variety of socioeconomic levels and environmental conditions. For this study, we also excluded 234 participants because of lack of genetic data (*n* = 136), lack of data on the status of excess weight (*n* = 10), lack of kin data (*n* = 61), and lack of data on food consumption (*n* = 27). According to these exclusions, our final sample consisted of 1211 children and adolescents.

### 2.2. Ethical Issues

The parents or legal guardians of each participating child signed an informed consent form in which the study procedures were described in detail. The study protocol was approved by the internal ethics committee of the Collective Health Institute of the Federal University of Bahia and by the National Research Ethics Council (CONEP) under references 003-05/CEP-ISC and 15.895/2011, respectively.

### 2.3. Data Collection

#### 2.3.1. Anthropometric Data

The weight was measured in grams and height in cm at the baseline. Weight was assessed using a portable electronic scale (Filizola^®^, model E-150/3P, São Paulo, Brazil), and height was measured with a portable stadiometer (Measure^®^Seca, Hamburg, Germany). Anthropometric measures were obtained according to standardized techniques performed by trained interviewers. To evaluate anthropometric status, the 2006 and 2007 reference tables of the World Health Organization (WHO), which are based on a *Z*-scores for body mass index (BMI) according to sex and age, were employed [17,18]. For statistical analyses, the excess weight was defined as follows: for children under 5 (*z* > +2 score), for children aged 5 to 10 years (*z* > +1), and for adolescents (*z* ≥ +1).

#### 2.3.2. DNA Extraction and Genotyping

DNA was extracted according to the protocol described by Gentra^®®^ Puregene^®®^ Blood Kit Qiagen (Germantown, ML, USA), while quantification was standardized at a concentration of 50 ng/μL and identified in barcoded tubes. This step was performed using a Qubit fluorometer^®^ (Invitrogen, Paisley, UK) [19]. The *LEPR* genetic information was extracted between positions 65,886,335 and 66,103,176 on chromosome 1 and genotyping for all 149 SNVs of the *LEPR* gene was conducted on the HumanOmni2.5-8 platform using the BeadChip kit (Illumina, San Diego, California, USA).

#### 2.3.3. Quality Control

A genetic quality control was performed before conducting the association tests. All procedures were performed with the help of PLINK v.1.9 (https://www.cog-genomics.org/plink/1.9/) [20]. To evaluate family structure, the kinship coefficients for each possible pair of this analysis were estimated. Sixty-one individuals were removed from the sample because of their degree of kinship. Quality control of the SNVs was performed in stages: by excluding SNVs with genotyping call rates under 0.98, a Hardy-Weinberg equilibrium (using only controls) with a *p*-value below 0.05, and a minor frequency allele (MAF) under 1% [21]. In candidate gene studies, the withdrawal of SNVs with very low frequency (1–2%) is common. To ensure the quality of the study, for small samples, it is recommended that MAF cut-off values be increased [22]. After the quality control, 112 SNVs were available for analysis.

#### 2.3.4. Population Structure

A principal component analysis (PCA) was performed to identify a possible population structure (population groups differentiated due to the ancestry/origin of each individual). Details on the PCA for population stratification are available in the work of Costa et al., 2015 [19].

#### 2.3.5. Food Consumption

Dietary intake was obtained through food recall for the last 24 h (24 h food recall—24 h) and a food frequency questionnaire (FFQ). Information was obtained from the parent or legal child’s guardian and collected by previously trained nutritionists and nutrition academics. Children over eight years of age also provided information on their food consumption outside of the home environment.

The 24-h diet-recall method (R24h) was used to determine dietary intake. Parents reported their children’s dietary intake. However, the information given by the children at the time of the interview complemented the information given by their parents. Food consumed in school or at day-care centers was also recorded. The food intake was converted into energy and macronutrient percentages using the Diet Pro program [23]. Foods that were not part of the software database were added using information contained in the Brazilian Food Composition Table [24], the Table for the Evaluation of Food Consumption from Home-Cooking Measurements [25], the ENDEF (*Estudo Nacional de Despesas Familiares*) food composition table [26], and packaged-food labels. The macronutrient intake was expressed as the percentage of the total energy intake. The ratio between polyunsaturated and saturated fat (POLY:SAT) was also considered.

Food consumption data were recorded in a semi-quantitative food frequency questionnaire (FFQ), validated by Matos et al. [27], consisting of 98 foods and related to food consumption in the last 12 months. Food frequency data were transformed into a daily intake fraction, to employ only one temporal unit. Subsequently, the foods were grouped on the basis of their nutritional characteristics [28]. The 11 food groups were as follows: Group 1—milk and dairy products (whole milk, yellow cheese, white cheese, yoghurt, fermented milk, heavy cream, other milk, skimmed milk); Group 2—processed meats (smoked meat, sausage/pepperoni, ham/mortadella); Group 3—Fried foods (churros, donuts, *pastel* (savory pastry), chips); Group 4—red meat (beef, sun-dried meat, viscera, pork); Group 5—refined cereals (rice, pasta, cake, biscuits, bread rolls, bread loafs, corn bread, oats, corn, cornmeal, white corn, pickled corn, corn flakes, corn starch); Group 6—legumes (boiled peanuts, roasted peanuts, textured soy, soymilk); Group 7—eggs (eggs); Group 8—chicken (chicken); Group 9—soft drinks/artificial juices (soft drinks and industrialized juices); Group 10—sweets (gelatin, jujube/lolly/marshmallow, ice lollies, ice cream, sweet popcorn, caramelized popcorn, chocolates, chocolate powder); and Group 11—margarine (margarine).

#### 2.3.6. Other Variables

The variables for adjustment were age, sex, population structure, and energy intake. The birth dates of the participants were obtained from their birth certificate, and the ages in years were calculated by subtracting the birth date from the interview date.

### 2.4. Statistical Analyses

The population was characterized by a descriptive analysis. The χ^2^ test was employed for categorical variables, and the Mann-Whitney U test was used to compare food intake variables of the “overweight/obese” and “not overweight/obese” groups.

Logistic regression analysis was used to evaluate the association between variants of the *LEPR* gene and overweightness. Each SNV of the *LEPR* gene was analyzed separately. Each *LEPR* gene SNV were determined using logistic regression models. Due to the low number of homozygotes for the risk allele, the genotype was analyzed using a dominant heredity genetic model adjusted for sex, age, population structure (determined by the first three major components), and energy intake. These confounding variables were selected on the basis of data published in previous studies [29,30].

Genomic and proteomic analyses regularly involve the simultaneous testing of hundreds of hypotheses in both numerical and categorical data. To correct for the occurrence of false positives, validation tests based on correction of multiple tests, such as Bonferroni and Benjamini, and Hochberg’s false discovery rate, and re-sampling techniques (permutation-based tests) are frequently employed. In this study, we used a permutation test because it has become a widely accepted and recommended approach for studies involving multiple statistical tests for genetic markers [31]. As such, empirical *p*-values were obtained after 50,000 phenotype permutations to limit the occurrence of type I errors (false-positive results). After the permutation tests, values of *p* < 0.05 were considered statistically significant.

Additional SNVs associated with overweightness/obesity in the SCAALA population were added to the interaction analyses, in addition to three *LEPR* SNVs (rs1137100, rs1137101, and rs8179183); these variants are commonly associated with excess weight, and have been frequently mentioned in the literature [8,32,33,34]. The interactions between *LEPR* gene SNVs and dietary intake were tested with respect to their effects on overweight/obesity, including the product terms in the models. The effect modification was analyzed by a likelihood-ratio test after estimation (lrtest).

To illustrate the interaction, the association between *LEPR* gene variants and excess weight was stratified by food consumption. To achieve this stratification, we considered calorie contribution percentages above or below the medians provided by carbohydrates, proteins, and total fat. The median daily intake frequencies of the 11 food groups were also the cut point for consideration in the analyses. The ratio between polyunsaturated and saturated fat (POLY:SAT) was also an independent variable.

All statistical tests were two-tailed, and the significance level considered was 5%. Statistical analyses were performed using PLINK version 1.9 [20] and STATA version 12.0. (Colllege Station, TX, USA).

## 3. Results

### 3.1. Characteristics of Participants and Dietary Intake

The eligible study population was 1445 children aged 4–11 years, 1211 of whom were included in the descriptive analysis. Of the children included in the study, 13.4% had excess weight (8.8% overweight, 4.6% obese). A slightly higher percentage was observed in boys compared with girls (53.8 vs. 46.2%). The median energy intake was 1651.78 (58.13–19307.12) Kcal. The median energy intake levels from carbohydrates, proteins, and fats were 61.58% (range: 27.08–87.38%), 12.60% (range: 4.28–82.69%), and 26.06% (range: 5.28–51.28%), respectively. The median intake of the polyunsaturated and saturated fat ratio (POLY/SAT) was 0.95 (range: 0.00–3.89). Significant differences were observed in total energy (*p* = 0.003), energy from protein (*p* = 0.034), and fat (*p* = 0.022) percentages between the groups studied (Table 1). The data also showed statistically significant differences in the median daily intake frequencies of milk and dairy products (*p* = 0.000), and processed meats (*p* = 0.042) groups amongst those who were overweight when compared with the non-overweight group. Additional information is provided in Table 2.

### 3.2. Association between LEPR Variants and Overweightness/Obesity

A genetic analysis, using a logistic regression model adjusted for sex, age, population structure, and energy intake, was conducted to evaluate the association between the *LEPR* gene variants and excess weight. There were four variants that were positively associated with overweightness/obesity: rs115650230 (G) (OR = 2.19; 95% CI = 1.08–4.45), rs116239759 (G) (OR = 2.25; 95% CI = 1.11–4.58), rs202069668 (C) (OR = 1.51; 95% CI = 1.07–2.13) and rs79353784 (A) (OR = 2.84; 95% CI = 1.19–6.75). However, the risk allele (G) of variant rs1177681 was shown to be at a borderline level of statistical significance for overweight/obesity (OR = 1.42; 95% CI = 1.00–2.00). The allele (G) of the rs78005150 variant was shown to be negatively associated (OR = 0.41; 95% CI = 0.17–0.95) with overweight/obesity (Table 3). Thus, the SNVs rs115650230, rs116239759, rs202069668, rs79353784, rs1177681, and rs78005150 in addition to SNVs rs1137100, rs1137101, and rs8179183, were included in the interaction analysis between the *LEPR* genotype and dietary intake, for a total of nine SNVs. The association between all *LEPR* gene SNVs genotyped from the SCAALA database and overweightness/obesity is given in a supplementary table (Supplementary Table S1).

### 3.3. The Role of Diet in the Association between LEPR Variants and Overweight/Obesity

An effect modification analysis of food intake regarding the association between *LEPR* gene variants and overweightness was performed (Table 4). An interaction was observed between fat intake (the ratio between PUFA:SFA) and the *LEPR* gene variant rs1137100 (interaction *p* = 0.049). The risk allele (G) for the rs1137100 variant was positively associated with overweightness/obesity in individuals whose POLY:SAT ratio was below the median (OR = 1.92; 95% CI = 1.18–3.14). Regarding the analyses run for the food groups, an interaction was found between daily soft drinks/artificial juices intake frequency and *LEPR* gene variant rs1137100 (interaction *p* = 0.019) relative to overweight/obesity. The risk allele (G) of the rs1137100 variant was positively associated with overweight/obesity in individuals whose daily soft drink/artificial juice intake frequency was above the median (OR = 2.15; 95% CI = 1.26–3.68). In addition, interactions between daily refined cereal intake frequency and *LEPR* gene variants rs1177681, rs1137100, and rs8179183 (interaction *p* < 0.001, 0.011 and 0.005, respectively) were observed. Positive associations were found between the risk alleles of the variants studied, and overweight/obesity in individuals whose daily refined cereal intake frequency was above the median: rs1177681 (G) (OR = 2.74; 95% CI = 1.65–4.57), rs1137100 (G) (OR = 2.17; 95% CI = 1.31–3.62). The risk allele (C) of variant rs8179183 was positively associated with overweightness/obesity in individuals whose refined cereal intake frequency was below the median (OR: 1.75; 95% CI: 1.06–2.90). Additional information is provided in Supplementary Tables S2 and S3.

## 4. Discussion

The prevalence of excess weight 13.4% (8.8% overweight, 4.6% obesity) in this study is lower than those recorded by the Family Budget Survey (POF) (*Pesquisa de Orçamento Familiar*) 2008–2009, in which the prevalence of overweightness fluctuated between the different regions of Brazil. Among children of 5 to 9 years, 33.5% were overweight and 14.3% were obese, and among adolescents aged 10–19 years, 20.5% were overweight and 4.9% were obese [1]. The criteria adopted for the diagnosis of overweight/obesity, the differences in the studied age groups and the cultural differences in each region may justify the variability observed in the studies. However, regardless of this variability, the prevalence of overweightness and obesity among children and adolescents in all macro-regions of the country, especially in the southeast, south, and center-west regions, stands out in general.

The value of this study derives in part from the characteristics of the population in which it was conducted. The population of Salvador is highly miscegenated. Lima-Costa et al. (2015) showed that 50% of the genetic composition of the Salvador-SCAALA cohort is of African origin [35] with the remainder being of European and Amerindian origin. For the *LEPR* gene from which the SNVs were evaluated, four variants were found to be positively associated with excess weight: rs115650230, rs116239759, rs202069668, and rs79353784. However, the rs78005150 variant showed a negative association with excess weight. Variants of the *LEPR* gene influencing obesity and obesity-related traits have been studied in children and adolescents in India [8], Spain [9], Mexico [36], [32] with the variants rs1137101, rs1137100, and rs8179183 being the most commonly studied. However, other studies, conducted with children of the same age group in Poland [12], Japan [37], Turkey [11], Malaysia [33], Denmark [34], and Brazil [38,39], failed to identify any significant association. Already in a study with young adults and Danish children [34], a negative association was observed. The heterogeneity between the results of association between overweightness/obesity, and different variants of the *LEPR* gene in different populations may depend on the ethnicity or the differences in the gene-environment interaction.

The role of diet in modulating the association between *LEPR* gene variants and overweight/obesity was examined through logistic regression analysis on food consumption in a well-characterized cohort of children and adolescents in Salvador, Bahia, Brazil. We found that a POLY:SAT ratio below the median potentiated the association between the risk allele of *LEPR* variant rs1137100 (G) (OR = 1.92; 95% CI = 1.18–3.14) and overweight/obesity. In contrast, a soft drinks/artificial juices intake frequency above the median maximized the obesogenic effect of the risk allele of variant rs1137100 (G) (OR = 2.15; 95% CI = 1.26–3.68). Similar results were observed when the intake frequency of the refined cereal group was above the median for SNVs rs1177681 (G) (OR = 2.74; 95% CI = 1.65–4.57), rs1137100 (G) (OR = 2.17; 95% CI = 1.31–3.62).

The literature offers few studies that evaluate the modifying effect of diet on the association between *LEPR* and overweightness/obesity, especially in children and adolescents. Dominguez-Reyes et al. [15] found an association in young Mexican individuals encoding alleles (AG + GG) for variant rs1137101 with saturated fat intake (SAT) ≥12 g/d (OR = 2.9; 95% CI = 1.5–5.8), and a greater risk of overweight/obesity when the total fat intake was ≥ 83 g/d (OR = 3.0; 95% CI = 1.5–6.2, *p* < 0.001). The beneficial effects of fatty acids, especially of polyunsaturated fatty acids (PUFA), on the modulation of the association between *LEPR* and overweight/obesity, has been noted by Jourdan et al. [40]. Likewise, the benefit of PUFA has been observed for polymorphisms of other genes, such as FTO [30,41], and studies have indicated an “anti-obesity” effect from PUFA. This finding is supported by the notion that unsaturated fats lead to greater diet-induced thermogenesis, energy expenditure, or fat oxidation, compared with saturated fatty acids (SFA) [42]. Multiple researchers have shown that fat oxidation is directly proportional to the PUFA:SFA ratio [43,44], which justifies the testing of this relationship. The mechanisms that allow dietary SFA to interact with *LEPR* are unknown; however, both the quality and quantity of dietary fat have been shown to influence the methylation of CpG regions [45]. Methylation consists of the addition of methyl group to cytosine (C), usually at CpG dinucleotides [46].

Our study also shows a significant interaction between higher soft drink/artificial juice intake frequency, and genetic predisposition related to overweight and obesity. The association between the frequent consumption of sugary beverages and overweightness in children and adults has been demonstrated in different prospective cohort studies, and randomized clinical trials that were reported in a systematic review and meta-analysis [47]. In addition to BMI and obesity, direct associations between sugary drink ingestion and waist circumference have been shown for these populations [48,49]. The ingestion of sugary drinks contributes to obesity through several potential mechanisms, including a high caloric content and reduced satiety. As liquids do not activate the inhibitory ingestion mechanisms in subsequent meals, increased total energy intake results [50]. Furthermore, the increasing portion sizes offered, combined with the presence of high concentrations of rapidly absorbed carbohydrates, such as sucrose or fructose-rich corn syrup, may increase the risk of visceral adiposity and other metabolic alterations [51]. Sugary drink ingestion may lead to increased genetic susceptibility to obesity, as seen from results obtained with three large cohorts in the United States of America [52]. These results were replicated in two other cohorts that showed an association between genetic variants with BMI and the incidence of obesity (based on 32 loci of GWAS associated with BMI) [53,54]. The effect was more pronounced amongst adult individuals with high sugary drink intake frequency, compared with those with infrequent consumption. In addition, an effect modification of these beverages on the association between genetic variants and abdominal obesity was found by Olsen et al. (2016) [55]. These and other findings suggest that the consumption of sweetened beverages potentiates the effect on weight gain in individuals who are genetically predisposed to storing body fat [53]; however, the mechanisms by which these relations take place are not clear. Studies involving the effect of these beverages on the association between the *LEPR* gene and weight gain in children and adolescents were not identified, which highlights the novelty of our findings.

This study showed an obesogenic effect from *LEPR* gene variants amongst those with increased refined cereal consumption frequency, which is a proxy for carbohydrates (CHO), with the exception of variant rs8179183. Although studies evaluating the modulating effects of specific carbohydrate sources in the relationship between *LEPR* and obesity were not identified, studies reporting the interactions between different genetic variants and carbohydrate intake are available [56,57]. Marti et al. (2006) [56] observed a higher risk between SNV pro12ala of the peroxisome proliferator-activated receptor gamma-gene (PPARy) and obesity (OR = 5.12, *p* < 0.04, 95% CI: 1.01–25.80) amongst individuals with higher CHO consumption (>49% energy). Likewise, Martinez et al. [57] observed that women with the Gln27Glu polymorphism of the β2-adrenergic receptor gene *(ADRB2)* with higher CHO intake (>49% energy) showed a higher risk of obesity (OR = 2.56, *p* = 0.051) than those with lower CHO intake, although with borderline statistical significance. Certainly, the cereal refinement process—which increases caloric density by 10%, reduces dietary fiber by 80%, and reduces the amount of dietary protein by almost 30%—may disqualify CHOs, and may be associated with insulin and leptin resistance, and body weight gain [58]. However, the genetic susceptibility to cereal-dependent weight gain still requires scientific investigation.

The literature shows that the leptin (*LEP*) and *LEPR* genes have been defined as a biological pathway for the regulation of food intake and energy expenditure [10]. For leptin to promote the appropriate neural response, it needs to bind to specific receptors (of the ObRb type) on the cell surface, promoting the activation of the JAK2/STAT3 system (Janus Kinase 2/signal transducer and activator of transcription 3), which regulates the synthesis of different neuropeptides that play important roles in the orexigenic and anorexigenic system involved in the control of food intake and energy balance [59]. Mutations in the leptin receptor gene compromise the transmission of the signal from the leptin binding to the receptor into the cell, and lead to leptin resistance, which is the reduced capacity of leptin to regulate appetite and weight gain [7].

Although the mechanisms involved in hypothalamic leptin resistance have been clarified, little is known about the effects of dietary components on the development of leptin resistance. Recently, new data have emerged from experimental animal studies indicating that specific types of dietary sugars or fats are capable of inducing leptin resistance in the absence of high levels of circulating leptin and/or body fat [60]. Shapiro et al. (2008) demonstrate that the dietary intake of fructose alone or in combination with high-fat diets contribute to obesity through two mechanisms: (1) induction of hyperphagia driven by increased palatability, and (2) blockade of leptin transport through the blood-brain barrier [61]. Despite advances in this field, studies dealing with this topic in human populations are still rare, and further studies are required to accurately define the role of the *LEPR* gene in the development of overweight and obesity.

Our study was based on an analysis of cross-sectional data, which limits the capacity to investigate causality. Furthermore, it was not possible to examine other adiposity measures, limiting this study to consider only BMI, which cannot distinguish body composition, and does not provide any indication of body fat distribution. The R24 h method employed in the present study to investigate food intake may also represent another limitation. Although the R24 h method is fast, relatively inexpensive, and easily applied, its success depends on the memory of the respondent and requires a well-trained investigator to obtain accurate estimates of the portions consumed. Nevertheless, this method provides reliable estimates of the average dietary intake of a population even when applied a single time, provided that the designated methodology is followed and the analytical resources are appropriate [62]. Moreover, most of the children included in this analysis are predominantly of African descent; therefore, our results cannot be generalized to other ethnic groups. In contrast, the strengths of this study include the interaction analysis with dietary intake and the analysis of a varied number of SNVs of the *LEPR* gene.

## 5. Conclusions

The association between *LEPR* and overweightness/obesity can be modified by the dietary characteristics, especially by the types and distributions of dietary fatty acids, sugary drinks, and refined cereals. The results of this study offer new insights into the interrelationships between the genetic variants of *LEPR*, and dietary intake and obesity. Nevertheless, further studies are needed to clarify the mechanisms involved in these relationships.

## Figures and Tables

**Table 1:** Characteristics of the study population. Salvador, Bahia, Brazil, 2005–2006.

**Variables**		**Total**	**Anthropometric Status**				
			**Not Overweight/Obesity**		**Overweight/Obesity**		**^a^*p* Value**
	***n***	**%**	**n**	**%**	**n**	**%**	
**Sex**							
Male	652	53.8	558	53.2	94	58	0.251
Female	559	46.2	491	46.8	68	42	
**Age**							
4–5	431	35.6	379	36.1	52	32.1	0.319
6–7	407	33.6	355	33.8	52	32.1	
8–11	373	30.8	315	30.0	58	35.8	
**Dietary Intake**	***n***	**Median** **(Min–Max)**	**Median** **(Min–Max)**		**Median** **(Min–Max)**		**^b^*p* Value**
Calorie (Kcal)	1211	1651.78 (58.13–19,307.12)	1616.15 (58.13–4812.15)		1771.29 (617.20–19,307.12)		0.003
Carbohydrate (% calories)	1211	61.58 (27.08–87.38)	61.73 (27.08–87.38)		60.53 (31.70–80.54)		0.277
Protein (% calories)	1211	12.60 (4.28–82.69)	12.54 (4.28–82.69)		13.16 (5.70–34.96)		0.034
Fat (% calories)	1211	26.06 (5.28–51.28)	25.83 (5.28–51.28)		27.27 (10.24–51.17)		0.022
POLY/SAT Ratio	1211	0.95 (0.00–3.89)	0.95 (0.00–3.89)		0.94 (0.06–3.66)		0.933

**^a^** Chi-square; **^b^** Mann-Whitney U.

**Table 2:** Food intake frequency according to food groups. Salvador, Bahia, Brazil, 2005–2006.

Variables	Total (*n* = 1211)		Anthropometric Status				
			Not Overweight/Obese		Overweight/Obese		*p* *
	n	%	n	%	n	%	
**Milk and dairy products**							
<median	636	52.5	572	54.5	64	39.5	0.000
≥median	575	47.5	477	45.5	98	60.5	
**Processed meats**							
<median	702	58.0	620	59.1	82	50.6	0.042
≥median	509	42.0	429	40.9	80	49.4	
**Fried foods**							
<median	531	43.8	461	43.9	70	43.2	0.860
≥median	680	56.2	588	56.1	92	56.8	
**Chicken**							
<median	769	63.5	677	64.5	92	56.8	0.057
≥median	442	36.5	372	35.5	70	43.2	
**Sweets**							
<median	602	49.7	517	49.3	85	52.5	0.451
≥median	609	50.3	532	50.7	77	47.5	
**Legumes**							
<median	785	64.8	676	64.4	109	67.3	0.481
≥median	426	35.2	373	35.6	53	32.7	
**Refined cereals**							
<median	599	49.5	512	48.8	87	53.7	0.246
≥median	612	50.5	537	51.2	75	46.3	
**Eggs**							
<median	1003	82.8	870	82.9	133	82.1	0.793
≥median	208	17.2	179	17.1	29	17.9	
**Meat**							
<median	593	49.0	520	49.6	73	45.1	0.285
≥median	618	51.0	529	50.4	89	54.9	
**Soft drinks/artificial juices**							
<median	711	58.7	617	58.8	94	58.0	0.849
≥median	500	41.3	432	41.2	68	42.0	
**Margarine**							
<median	443	36.6	379	36.1	64	39.5	0.406
≥median	768	63.4	670	63.9	98	60.5	

*p* *—Chi-square test.

**Table 3:** Logistic regression between variants of *LEPR* and excess weight. Salvador, Bahia, Brazil, 2005–2006.

SNVs	Risk Allele	MAF	OR	95% CI		*p* *
rs1137100	G	0.17	1.36	0.95	1.95	0.096
rs1137101	G	0.48	0.94	0.64	1.38	0.757
rs115650230	G	0.02	2.19	1.08	4.45	0.018
rs116239759	G	0.02	2.25	1.11	4.58	0.014
rs1177681	G	0.20	1.42	1.00	2.00	0.050
rs202069668	C	0.28	1.51	1.07	2.13	0.019
rs78005150	G	0.04	0.41	0.17	0.95	0.039
rs79353784	A	0.01	2.84	1.19	6.75	0.016
rs8179183	C	0.21	1.06	0.74	1.52	0.752

OR—Dominant model adjusted by gender, age, energy, PC1, PC2, PC3. * *p*: 50,000 permutations, adjusted for energy, gender, age, PC1, PC2, P3.

**Table 4:** Association between *LEPR* gene variants and excess weight according to food consumption. Salvador, Bahia, Brazil, 2005–2006.

SNVs		Phenotypes	OR	95% CI	*p* Interaction*
**Dietary PUFA:SFA**					
rs1137100	<median	Excess weight	1.92	1.18–3.14	0.049
	≥median	Excess weight	0.94	0.55–1.60	
**Soft Drinks/Artificial Juices**					
rs1137100	<median	Excess weight	0.91	0.55–1.50	0.019
	≥median	Excess weight	2.15	1.26–3.68	
**Refined cereal**					
rs1177681	<median	Excess weight	0.75	0.45–1.25	0.000
	≥median	Excess weight	2.74	1.65–4.57	
rs1137100	<median	Excess weight	0.84	0.50–1.42	0.011
	≥median	Excess weight	2.17	1.31–3.62	
rs8179183	<median	Excess weight	1.75	1.06–2.90	0.005
	≥median	Excess weight	0.63	0.35–1.12	

* *p*—Interaction test: likelihood ratio adjusted by sex, age, energy, PC1, PC2, and PC3. OR—Dominant model adjusted by sex, age, energy, PC1, PC2, and PC3.

## Supplementary Materials

The following are available online at http://www.mdpi.com/2072-6643/10/8/1117/s1, Table S1: Logistic regression between variants of *LEPR* and excess weight. Salvador, Bahia, Brazil, 2005–2006, Table S2: Association between *LEPR* gene variants and excess weight according to dietary intake. Salvador, Bahia, Brazil, 2005–2006, Table S3: Association between *LEPR* gene variants and excess weight according to food group. Salvador, Bahia, Brazil, 2005–2006.

## References

[1] Brasil (2010). Pesquisa de Orçamentos Familiares: 2008–2009. Antropometria e Estado Nutricional de Crianças, Adolescentes e Adultos no Brasil.

[2] Foraita R., Gunther F., Gwozdz W., Reisch L.A., Russo P., Lauria F., Siani A., Veidebaum T., Tornaritis M., Iacoviello L. (2015). Does the FTO gene interact with the socioeconomic status on the obesity development among young European children? Results from the IDEFICS study. Int. J. Obes..

[3] Perez L.M., Garcia K., Herrera R. (2013). Psychological, behavioral and familial factors in obese Cuban children and adolescents. MEDICC Rev..

[4] World of Health Organization (2004). Global Strategy on Diet, Physical Activity and Health.

[5] Srivastava A., Srivastava N., Mittal B. (2015). Genetics of Obesity. Indian J. Clin. Biochem..

[6] Farooqi I.S., O’Rahilly S. (2014). 20 years of leptin: Human disorders of leptin action. J. Endocrinol..

[7] Jequier E. (2002). Leptin signaling, adiposity, and energy balance. Ann. N. Y. Acad. Sci..

[8] Tabassum R., Mahendran Y., Dwivedi O.P., Chauhan G., Ghosh S., Marwaha R.K., Tandon N., Bharadwaj D. (2012). Common variants of IL6, *LEPR*, and PBEF1 are associated with obesity in Indian children. Diabetes.

[9] Riestra P., Garcia-Anguita A., Schoppen S., Lopez-Simon L., De Oya M., Garces C. (2010). Sex-specific association between leptin receptor polymorphisms and leptin levels and BMI in healthy adolescents. Acta Paediatr..

[10] Marginean C.O., Marginean C., Voidazan S., Melit L., Crauciuc A., Duicu C., Banescu C. (2016). Correlations between Leptin Gene Polymorphisms 223 A/G, 1019 G/A, 492 G/C, 976 C/A, and Anthropometrical and Biochemical Parameters in Children with Obesity: A Prospective Case-Control Study in a Romanian Population—The Nutrichild Study. Medicine.

[11] Komsu-Ornek Z., Demirel F., Dursun A., Ermis B., Piskin E., Bideci A. (2012). Leptin receptor gene Gln223Arg polymorphism is not associated with obesity and metabolic syndrome in Turkish children. Turk. J. Pediatr..

[12] Pyrzak B., Wisniewska A., Kucharska A., Wasik M., Demkow U. (2009). No association of *LEPR* Gln223Arg polymorphism with leptin, obesity or metabolic disturbances in children. Eur. J. Med. Res..

[13] Qi L., Cho Y.A. (2008). Gene-environment interaction and obesity. Nutr. Rev..

[14] Ordovas J.M. (2006). Nutrigenetics, plasma lipids, and cardiovascular risk. J. Am. Diet. Assoc..

[15] Dominguez-Reyes T., Astudillo-Lopez C.C., Salgado-Goytia L., Munoz-Valle J.F., Salgado-Bernabe A.B., Guzman-Guzman I.P., Castro-Alarcon N., Moreno-Godinez M.E., Parra-Rojas I. (2015). Interaction of dietary fat intake with APOA2, APOA5 and *LEPR* polymorphisms and its relationship with obesity and dyslipidemia in young subjects. Lipids Health Dis..

[16] Barreto M.L., Cunha S.S., Alcântara-Neves N., Carvalho L.P., Cruz A.A., Stein R.T., Genser B., Cooper P.J., Rodrigues L.C. (2006). Risk factors and immunological pathways for asthma and other allergic diseases in children: Background and methodology of a longitudinal study in a large urban center in Northeastern Brazil (Salvador-SCAALA study). BMC Pulm. Med..

[17] World Health Organization (2007). Child Growth Standards: Head Circumference-for-Age, Arm Circumference-for-Age, Triceps Skinfold-for-Age and Subscapular Skinfold-for-Age. Methods and Development.

[18] World Health Organization (2006). Child Growth Standards: Length/Height-for-Age, Weight-for-Age, Weight-for-Length, Weight-for-Height and Body Mass Index-for-Age. Methods and Development.

[19] Costa G.N., Dudbridge F., Fiaccone R.L., da Silva T.M., Conceicao J.S., Strina A., Figueiredo C.A., Magalhaes W.C., Rodrigues M.R., Gouveia M.H. (2015). A genome-wide association study of asthma symptoms in Latin American children. BMC Genet..

[20] Chang C.C., Chow C.C., Tellier L.C., Vattikuti S., Purcell S.M., Lee J.J. (2015). Second-generation PLINK: Rising to the challenge of larger and richer datasets. Gigascience.

[21] Laurie C.C., Doheny K.F., Mirel D.B., Pugh E.W., Bierut L.J., Bhangale T., Boehm F., Caporaso N.E., Cornelis M.C., Edenberg H.J. (2010). Quality control and quality assurance in genotypic data for genome-wide association studies. Genet. Epidemiol..

[22] Anderson C.A., Pettersson F.H., Clarke G.M., Cardon L.R., Morris A.P., Zondervan K.T. (2010). Data quality control in genetic case-control association studies. Nat. Protoc..

[23] (2006). DietPro, Versão 4.0, Sistema de Análise Nutricional.

[24] Núcleo de Estudos e Pesquisas em Alimentação (2006). Tabela de Composicão de Alimentos.

[25] Pinheiro A.B.V., Lacerda E.M.D.A., Benzecry E.H., Gomes M.C.D.S., Costa V.M.D. (2004). Tabela para Avaliação de Consumo Alimentar em Medidas Caseiras.

[26] ENDEF (1996). Tabela de Composição de Alimentos. Fundacao Instituto Brasileiro de Geografia e Estatística.

[27] Matos S.M., Prado M.S., Santos C.A., D’Innocenzo S., Assis A.M., Dourado L.S., Oliveira N.S., Rodrigues L.C., Barreto M.L. (2012). Validation of a food frequency questionnaire for children and adolescents aged 4 to 11 years living in Salvador, Bahia. Nutr. Hosp..

[28] D’Innocenzo S., Marchioni D.M.L., Prado M.S., Matos S.M.A., Pereira S.R.S., Barros A.P., Sampaio L.R., Assis A.M.O., Rodrigues L.C., Barreto M.L. (2011). Condições socioeconômicas e padrões alimentares de crianças de 4 a 11 anos: Estudo SCAALA—Salvador/Bahia. Rev. Bras. Saúde Matern. Infant..

[29] Corella D., Lai C.Q., Demissie S., Cupples L.A., Manning A.K., Tucker K.L., Ordovas J.M. (2007). APOA5 gene variation modulates the effects of dietary fat intake on body mass index and obesity risk in the Framingham Heart Study. J. Mol. Med..

[30] Vilella M., Nunes de Oliveira Costa G., Lima Barreto M., Alexandrina Figueredo C., Maria Alcantara-Neves N., Cunha Rodrigues L., Maria Alvim de Matos S., Leovigildo Fiaccone R., Oliveira P., Rocha A. (2017). Effect of dietary consumption as a modifier on the association between FTO gene variants and excess body weight in children from an admixed population in Brazil: The Social Changes, Asthma and Allergy in Latin America (SCAALA) cohort study. Br. J. Nutr..

[31] Belmonte M., Yurgelun-Todd D. (2001). Permutation testing made practical for functional magnetic resonance image analysis. IEEE Trans. Med. Imaging.

[32] Angel-Chavez L.I., Tene-Perez C.E., Castro E. (2012). Leptin receptor gene K656N polymorphism is associated with low body fat levels and elevated high-density cholesterol levels in Mexican children and adolescents. Endocr. Res..

[33] Ng Z.Y., Veerapen M.K., Hon W.M., Lim R.L. (2014). Association of leptin/receptor and TNF-alpha gene variants with adolescent obesity in Malaysia. Pediatr. Int..

[34] Hollensted M., Ahluwalia T.S., Have C.T., Grarup N., Fonvig C.E., Nielsen T.R., Trier C., Paternoster L., Pedersen O., Holm J.C. (2015). Common variants in LEPR, IL6, AMD1, and NAMPT do not associate with risk of juvenile and childhood obesity in Danes: A case-control study. BMC Med. Genet..

[35] Lima-Costa M.F., Rodrigues L.C., Barreto M.L., Gouveia M., Horta B.L., Mambrini J., Kehdy F.S., Pereira A., Rodrigues-Soares F., Victora C.G. (2015). Genomic ancestry and ethnoracial self-classification based on 5871 community-dwelling Brazilians (The Epigen Initiative). Sci. Rep..

[36] Guizar-Mendoza J.M., Amador-Licona N., Flores-Martinez S.E., Lopez-Cardona M.G., Ahuatzin-Tremary R., Sanchez-Corona J. (2005). Association analysis of the Gln223Arg polymorphism in the human leptin receptor gene, and traits related to obesity in Mexican adolescents. J. Hum. Hypertens..

[37] Endo K., Yanagi H., Hirano C., Hamaguchi H., Tsuchiya S., Tomura S. (2000). Association of Trp64Arg polymorphism of the beta3-adrenergic receptor gene and no association of Gln223Arg polymorphism of the leptin receptor gene in Japanese schoolchildren with obesity. Int. J. Obes. Relat. Metab. Disord..

[38] Queiroz E.M., Candido A.P., Castro I.M., Bastos A.Q., Machado-Coelho G.L., Freitas R.N. (2015). IGF2, LEPR, POMC, PPARG, and PPARGC1 gene variants are associated with obesity-related risk phenotypes in Brazilian children and adolescents. Braz. J. Med. Biol. Res..

[39] Zandoná M.R., Rodrigues R.O., Albiero G., Dal P., Campagnolo B., Vitolo M.R., Almeida S., Mattevi V.S. (2013). Polymorphisms in LEPR, PPARG and APM1 genes: Associations with energy intake and metabolic traits in young children. Arq. Bras. Endocrinol. Metab..

[40] Jourdan C., Kloiber S., Nieters A., Seiler H., Himmerich H., Kohli M.A., Lucae S., Wolfram G., Gieger C., Wichmann H.E. (2011). Gene-PUFA interactions and obesity risk. Br. J. Nutr..

[41] Corella D., Carrasco P., Sorl J.V., Coltell O., Ortega-Azorín C., Guillén M., González J.I., Sáiz C., Estruch R., Ordovas J.M. (2012). Education modulates the association of the FTO rs9939609 polymorphism with body mass index and obesity risk in the Mediterranean population. Nutr. Metab. Cardiovasc. Dis..

[42] Krishnan S., Cooper J.A. (2014). Effect of dietary fatty acid composition on substrate utilization and body weight maintenance in humans. Eur. J. Nutr..

[43] Seaton T.B., Welle S.L., Warenko M.K., Campbell R.G. (1986). Thermic effect of medium-chain and long-chain triglycerides in man. Am. J. Clin. Nutr..

[44] DeLany J.P., Windhauser M.M., Champagne C.M., Bray G.A. (2000). Differential oxidation of individual dietary fatty acids in humans. Am. J. Clin. Nutr..

[45] Voisin S., Almen M.S., Moschonis G., Chrousos G.P., Manios Y., Schioth H.B. (2015). Dietary fat quality impacts genome-wide DNA methylation patterns in a cross-sectional study of Greek preadolescents. Eur. J. Hum. Genet..

[46] Hashimshony T., Zhang J., Keshet I., Bustin M., Cedar H. (2003). The role of DNA methylation in setting up chromatin structure during development. Nat. Genet..

[47] Malik V.S., Pan A., Willett W.C., Hu F.B. (2013). Sugar-sweetened beverages and weight gain in children and adults: A systematic review and metaanalysis. Am. J. Clin. Nutr..

[48] Collison K.S., Zaidi M.Z., Subhani S.N., Al-Rubeaan K., Shoukri M., Al-Mohanna F.A. (2010). Sugar-sweetened carbonated beverage consumption correlates with BMI, waist circumference, and poor dietary choices in school children. BMC Public Health.

[49] Odegaard A.O., Choh A.C., Czerwinski S.A., Towne B., Demerath E.W. (2012). Sugar-sweetened and diet beverages in relation to visceral adipose tissue. Obesity.

[50] Almiron-Roig E., Palla L., Guest K., Ricchiuti C., Vint N., Jebb S.A., Drewnowski A. (2013). Factors that determine energy compensation: A systematic review of preload studies. Nutr. Rev..

[51] Stanhope K.L., Schwarz J.M., Keim N.L., Griffen S.C., Bremer A.A., Graham J.L., Hatcher B., Cox C.L., Dyachenko A., Zhang W. (2009). Consuming fructose-sweetened, not glucose-sweetened, beverages increases visceral adiposity and lipids and decreases insulin sensitivity in overweight/obese humans. J. Clin. Investig..

[52] Qi Q., Downer M.K., Kilpelainen T.O., Taal H.R., Barton S.J., Ntalla I., Standl M., Boraska V., Huikari V., Kiefte-De Jong J.C. (2015). Dietary intake, FTO genetic variants, and adiposity: A combined analysis of over 16,000 children and adolescents. Diabetes.

[53] Brunkwall L., Chen Y., Hindy G., Rukh G., Ericson U., Barroso I., Johansson I., Franks P.W., Orho-Melander M., Renstrom F. (2016). Sugar-sweetened beverage consumption and genetic predisposition to obesity in 2 Swedish cohorts. Am. J. Clin. Nutr..

[54] Qi Q., Chu A.Y., Kang J.H., Jensen M.K., Curhan G.C., Pasquale L.R., Ridker P.M., Hunter D.J., Willett W.C., Rimm E.B. (2012). Sugar-sweetened beverages and genetic risk of obesity. N. Engl. J. Med..

[55] Olsen N.J., Ängquist L., Larsen S.C., Linneberg A., Skaaby T., Husemoen L.L., Toft U., Tjønneland A., Halkjær J., Hansen T. (2016). Interactions between genetic variants associated with adiposity traits and soft drinks in relation to longitudinal changes in body weight and waist circumference. Am. J. Clin. Nutr..

[56] Marti A.C.M., Martínez-González M.A., Forga L., Martínez J.A. (2002). CHO intake alters obesity risk associated with Pro12Ala polymorphism of PPARgamma gene. J. Physiol. Biochem..

[57] Martínez J.A., Corbalán M.S., Sánchez-Villegas A., Forga L., Marti A., Martínez-González M.A. (2003). Obesity Risk Is Associated with Carbohydrate Intake in Women Carrying the Gln27Glu β2-Adrenoceptor Polymorphism. J. Nutr..

[58] Durtschi A. (2001). Three patients’ tele-home care experiences. Home Heal. Nurse.

[59] Fruhbeck G. (2006). Intracellular signalling pathways activated by leptin. Biochem. J..

[60] Vasselli J.R., Scarpace P.J., Harris R.B., Banks W.A. (2013). Dietary components in the development of leptin resistance. Adv. Nutr..

[61] Shapiro A., Mu W., Roncal C., Cheng K.Y., Johnson R.J., Scarpace P.J. (2008). Fructose-induced leptin resistance exacerbates weight gain in response to subsequent high-fat feeding. Am. J. Physiol. Regul. Integr. Comp. Physiol..

[62] Willett W., Stampfer M.J. (1986). Total energy intake: Implications for epidemiologic analyses. Am. J. Epidemiol..

