# Supplementary material for: Food Consumption as a Modifier of the Association between LEPR Gene Variants and Excess Body Weight in Children and Adolescents: A Study of the SCAALA Cohort
Source: Nutrients. 2018 Aug 18;10(8):1117. doi: 10.3390/nu10081117 (PMC6116060; doi:10.3390/nu10081117)
Supplement: Supplementary file 1 [file nutrients-10-01117-s001.zip › Supplementary table 1.docx]

**Supplementary table 1:** Logistic regression between variants of *LEPR* and excess weight. Salvador, Bahia, Brazil, 2005-2006.

| SNVs | Position | Genotype | Not excess weight | excess weight | OR | 95% CI | | p_value_ | p* |
| --- | --- | --- | --- | --- | --- | --- | --- | --- | --- |
| rs10157915 | 66031528 | AA | 979 | 149 | 1.31 | 0.73 | 2.35 | 0.368 | 0.343 |
|  |  | AT+TT | 89 | 16 |  |  |  |  |  |
| rs10158279 | 66033696 | TT | 273 | 41 | 1.04 | 0.70 | 1.54 | 0.859 | 0.859 |
|  |  | TG+GG | 800 | 124 |  |  |  |  |  |
| rs1045895 | 65897981 | GG | 685 | 63 | 1.12 | 0.79 | 1.59 | 0.534 | 0.536 |
|  |  | GA+AA | 387 | 102 |  |  |  |  |  |
| rs1046011 | 65898996 | CC | 664 | 104 | 1.04 | 0.73 | 1.49 | 0.816 | 0.815 |
|  |  | CT+ TT | 408 | 60 |  |  |  |  |  |
| rs10493380 | 66046117 | AA | 791 | 122 | 1.00 | 0.67 | 1.49 | 0.997 | 0.998 |
|  |  | AC+CC | 278 | 43 |  |  |  |  |  |
| rs10749753 | 65998467 | AA | 418 | 67 | 0.99 | 0.70 | 1.41 | 0.962 | 0.963 |
|  |  | AG+GG | 655 | 98 |  |  |  |  |  |
| rs10889557 | 65970704 | GG | 499 | 73 | 1.08 | 0.76 | 1.53 | 0.658 | 0.663 |
|  |  | GA+AA | 570 | 91 |  |  |  |  |  |
| rs10889558 | 65976966 | GG | 528 | 80 | 1.02 | 0.72 | 1.44 | 0.916 | 0.917 |
|  |  | GA+AA | 545 | 85 |  |  |  |  |  |
| rs111291360 | 65982699 | AA | 1020 | 156 | 1.22 | 0.58 | 2.55 | 0.601 | 0.597 |
|  |  | AC+CC | 53 | 9 |  |  |  |  |  |
| rs11208648 | 65896612 | AA | 644 | 96 | 0.94 | 0.66 | 1.34 | 0.739 | 0.741 |
|  |  | AG+GG | 429 | 69 |  |  |  |  |  |
| rs11208654 | 65965566 | TT | 627 | 92 | 1.23 | 0.87 | 1.73 | 0.246 | 0.246 |
|  |  | TC+CC | 455 | 73 |  |  |  |  |  |
| rs11208659 | 65979280 | TT | 576 | 85 | 1.03 | 0.73 | 1.45 | 0.877 | 0.875 |
|  |  | TC+CC | 497 | 80 |  |  |  |  |  |
| rs11208660 | 65983626 | CC | 756 | 114 | 1.05 | 0.72 | 1.51 | 0.812 | 0.808 |
|  |  | CT+TT | 317 | 51 |  |  |  |  |  |
| rs11208664 | 66000605 | TT | 1029 | 160 | 0.64 | 0.19 | 2.19 | 0.475 | 0.480 |
|  |  | TC+CC | 34 | 4 |  |  |  |  |  |
| rs11208675 | 66045424 | TT | 600 | 84 | 1.26 | 0.90 | 1.78 | 0.183 | 0.183 |
|  |  | TG+GG | 473 | 81 |  |  |  |  |  |
| rs1137100 | 66036441 | AA | 744 | 105 | 1.36 | 0.95 | 1.94 | 0.094 | 0.096 |
|  |  | AG+GG | 323 | 60 |  |  |  |  |  |
| rs1137101 | 66058513 | AA | 282 | 46 | 0.94 | 0.64 | 1.38 | 0.758 | 0.757 |
|  |  | AG+GG | 791 | 119 |  |  |  |  |  |
| rs114270433 | 65894690 | TT | 1002 | 160 | 0.46 | 0.18 | 1.17 | 0.102 | 0.087 |
|  |  | TC+CC | 71 | 5 |  |  |  |  |  |
| rs114315471 | 66045329 | AA | 1027 | 155 | 1.67 | 0.77 | 3.60 | 0.191 | 0.158 |
|  |  | AG+GG | 40 | 10 |  |  |  |  |  |
| rs114505530 | 65890919 | CC | 1012 | 159 | 0.47 | 0.17 | 1.34 | 0.158 | 0.124 |
|  |  | CT+TT | 57 | 5 |  |  |  |  |  |
| rs115606451 | 66098007 | AA | 1035 | 164 | 0.27 | 0.04 | 2.04 | 0.205 | 0.131 |
|  |  | AG+GG | 25 | 1 |  |  |  |  |  |
| rs115650230 | 65945430 | AA | 1038 | 154 | 2.19 | 1.08 | 4.45 | **0.030** | **0.018** |
|  |  | AG+GG | 35 | 11 |  |  |  |  |  |
| rs11579567 | 65957141 | CC | 825 | 128 | 0.83 | 0.55 | 1.26 | 0.383 | 0.384 |
|  |  | CA+AA | 244 | 37 |  |  |  |  |  |
| rs11585329 | 66073814 | GG | 919 | 137 | 1.23 | 0.78 | 1.96 | 0.372 | 0.376 |
|  |  | GT+TT | 154 | 28 |  |  |  |  |  |
| rs116239759 | 65995492 | AA | 1037 | 154 | 2.25 | 1.11 | 4.58 | **0.025** | **0.014** |
|  |  | AG+GG | 36 | 11 |  |  |  |  |  |
| rs116320511 | 65948353 | AA | 995 | 154 | 0.78 | 0.38 | 1.61 | 0.503 | 0.500 |
|  |  | AG+GG | 78 | 11 |  |  |  |  |  |
| rs1171269 | 65996802 | CC | 694 | 101 | 1.17 | 0.82 | 1.66 | 0.388 | 0.387 |
|  |  | CT+TT | 379 | 64 |  |  |  |  |  |
| rs1177681 | 65999119 | AA | 707 | 98 | 1.42 | 1.00 | 2.00 | 0.051 | 0.050 |
|  |  | AG+GG | 363 | 68 |  |  |  |  |  |
| rs11801408 | 66075136 | CC | 462 | 73 | 0.90 | 0.64 | 1.26 | 0.532 | 0.529 |
|  |  | CT+TT | 610 | 92 |  |  |  |  |  |
| rs11808888 | 65952781 | GG | 418 | 72 | 0.79 | 0.56 | 1.11 | 0.177 | 0.176 |
|  |  | GA+AA | 654 | 93 |  |  |  |  |  |
| rs12038998 | 66049128 | CC | 585 | 84 | 1.22 | 0.86 | 1.72 | 0.259 | 0.262 |
|  |  | CA+AA | 485 | 81 |  |  |  |  |  |
| rs12042877 | 66053148 | CC | 608 | 92 | 1.06 | 0.75 | 1.50 | 0.724 | 0.726 |
|  |  | CT+TT | 465 | 73 |  |  |  |  |  |
| rs12059300 | 66047072 | GG | 674 | 114 | 0.74 | 0.51 | 1.07 | 0.113 | 0.111 |
|  |  | GA+AA | 399 | 51 |  |  |  |  |  |
| rs12065099 | 65896308 | TT | 641 | 96 | 0.93 | 0.66 | 1.32 | 0.697 | 0.695 |
|  |  | TC+CC | 432 | 69 |  |  |  |  |  |
| rs12135802 | 65992140 | AA | 918 | 147 | 0.68 | 0.40 | 1.18 | 0.170 | 0.172 |
|  |  | AG+GG | 155 | 18 |  |  |  |  |  |
| rs12140910 | 66002660 | AA | 953 | 150 | 0.72 | 0.40 | 1.31 | 0.277 | 0.281 |
|  |  | AG+GG | 120 | 15 |  |  |  |  |  |
| rs12145690 | 65887013 | AA | 350 | 53 | 1.02 | 0.70 | 1.46 | 0.938 | 0.939 |
|  |  | AC+CC | 723 | 112 |  |  |  |  |  |
| rs12409877 | 65943872 | GG | 286 | 42 | 1.07 | 0.72 | 1.58 | 0.749 | 0.751 |
|  |  | GA+AA | 786 | 123 |  |  |  |  |  |
| rs1327115 | 65966839 | GG | 418 | 56 | 1.27 | 0.89 | 1.83 | 0.193 | 0.193 |
|  |  | GT+TT | 655 | 109 |  |  |  |  |  |
| rs1327121 | 65957337 | TT | 622 | 90 | 1.26 | 0.89 | 1.78 | 0.188 | 0.187 |
|  |  | TC+CC | 446 | 74 |  |  |  |  |  |
| rs1475397 | 65983158 | TT | 333 | 47 | 1.19 | 0.82 | 1.74 | 0.361 | 0.363 |
|  |  | TC+CC | 740 | 118 |  |  |  |  |  |
| rs17097193 | 66067396 | TT | 879 | 130 | 1.20 | 0.78 | 1.83 | 0.407 | 0.404 |
|  |  | TC+CC | 187 | 34 |  |  |  |  |  |
| rs17127608 | 65893941 | CC | 890 | 137 | 0.90 | 0.56 | 1.43 | 0.651 | 0.655 |
|  |  | CT+TT | 183 | 28 |  |  |  |  |  |
| rs17127655 | 65935207 | CC | 845 | 130 | 0.88 | 0.57 | 1.36 | 0.558 | 0.552 |
|  |  | CT+TT | 227 | 35 |  |  |  |  |  |
| rs17127669 | 65952711 | TT | 1002 | 155 | 0.80 | 0.37 | 1.72 | 0.562 | 0.572 |
|  |  | TC+CC | 65 | 10 |  |  |  |  |  |
| rs17127826 | 66094550 | AA | 766 | 121 | 0.82 | 0.55 | 1.21 | 0.313 | 0.316 |
|  |  | AG+GG | 304 | 44 |  |  |  |  |  |
| rs17415296 | 66099013 | CC | 820 | 123 | 1.10 | 0.74 | 1.64 | 0.633 | 0.634 |
|  |  | CA+AA | 253 | 42 |  |  |  |  |  |
| rs1751490 | 66007430 | CC | 503 | 71 | 1.18 | 0.84 | 1.67 | 0.342 | 0.342 |
|  |  | CA+AA | 563 | 93 |  |  |  |  |  |
| rs1805134 | 66067109 | TT | 532 | 87 | 0.89 | 0.63 | 1.25 | 0.489 | 0.488 |
|  |  | TC+CC | 534 | 77 |  |  |  |  |  |
| rs1887285 | 65897747 | TT | 860 | 126 | 1.28 | 0.86 | 1.92 | 0.229 | 0.227 |
|  |  | TC+CC | 210 | 39 |  |  |  |  |  |
| rs1938489 | 66048622 | AA | 628 | 101 | 0.89 | 0.63 | 1.27 | 0.527 | 0.530 |
|  |  | AG+GG | 438 | 63 |  |  |  |  |  |
| rs200163763 | 66001360 | AA | 1032 | 159 | 0.97 | 0.40 | 2.35 | 0.946 | 0.858 |
|  |  | AC+CC | 39 | 6 |  |  |  |  |  |
| rs202069668 | 65996650 | TT | 588 | 75 | 1.51 | 1.07 | 2.13 | **0.020** | **0.019** |
|  |  | TC+ CC | 485 | 90 |  |  |  |  |  |
| rs2025803 | 65946506 | TT | 638 | 97 | 1.07 | 0.76 | 1.52 | 0.696 | 0.695 |
|  |  | TC+CC | 432 | 67 |  |  |  |  |  |
| rs2154380 | 66057441 | AA | 284 | 46 | 0.95 | 0.65 | 1.40 | 0.795 | 0.794 |
|  |  | AG+GG | 789 | 119 |  |  |  |  |  |
| rs2376016 | 66033996 | CC | 665 | 107 | 0.85 | 0.59 | 1.22 | 0.370 | 0.369 |
|  |  | CT+TT | 400 | 55 |  |  |  |  |  |
| rs34499590 | 66081791 | CC | 1024 | 158 | 1.00 | 0.38 | 2.67 | 0.994 | 0.998 |
|  |  | CT+TT | 35 | 6 |  |  |  |  |  |
| rs36072366 | 65890236 | TT | 315 | 57 | 0.79 | 0.55 | 1.14 | 0.211 | 0.212 |
|  |  | TC+CC | 756 | 108 |  |  |  |  |  |
| rs3790433 | 65894342 | AA | 332 | 51 | 1.12 | 0.77 | 1.62 | 0.563 | 0.566 |
|  |  | AG+GG | 741 | 114 |  |  |  |  |  |
| rs4468199 | 65893960 | AA | 814 | 119 | 1.16 | 0.79 | 1.71 | 0.453 | 0.453 |
|  |  | AG+GG | 258 | 45 |  |  |  |  |  |
| rs4567312 | 66089582 | CC | 886 | 139 | 0.71 | 0.44 | 1.17 | 0.180 | 0.180 |
|  |  | CT+TT | 187 | 26 |  |  |  |  |  |
| rs4606347 | 66073361 | GG | 677 | 101 | 1.07 | 0.75 | 1.52 | 0.728 | 0.723 |
|  |  | GA+AA | 389 | 64 |  |  |  |  |  |
| rs4655537 | 66058801 | GG | 403 | 63 | 1.01 | 0.71 | 1.44 | 0.955 | 0.955 |
|  |  | GA+AA | 669 | 102 |  |  |  |  |  |
| rs4655802 | 65888231 | AA | 379 | 67 | 0.81 | 0.57 | 1.15 | 0.247 | 0.243 |
|  |  | AG+GG | 694 | 98 |  |  |  |  |  |
| rs55730790 | 65890397 | AA | 820 | 120 | 1.32 | 0.90 | 1.94 | 0.160 | 0.160 |
|  |  | AG+GG | 250 | 45 |  |  |  |  |  |
| rs57669574 | 66084569 | CC | 872 | 142 | 0.66 | 0.40 | 1.07 | 0.094 | 0.094 |
|  |  | CT+TT | 201 | 23 |  |  |  |  |  |
| rs57792498 | 65892709 | GG | 685 | 102 | 0.98 | 0.69 | 1.40 | 0.915 | 0.915 |
|  |  | GA+AA | 387 | 63 |  |  |  |  |  |
| rs58382953 | 66063309 | GG | 1007 | 154 | 0.98 | 0.48 | 2.01 | 0.956 | 0.938 |
|  |  | GA+AA | 66 | 11 |  |  |  |  |  |
| rs61779781 | 65934354 | TT | 684 | 104 | 1.11 | 0.78 | 1.59 | 0.553 | 0.556 |
|  |  | TC+CC | 389 | 61 |  |  |  |  |  |
| rs61781284 | 66054038 | GG | 764 | 121 | 0.89 | 0.60 | 1.32 | 0.560 | 0.557 |
|  |  | GA+AA | 307 | 44 |  |  |  |  |  |
| rs6413506 | 66102224 | AA | 999 | 156 | 0.86 | 0.41 | 1.77 | 0.675 | 0.646 |
|  |  | AG+GG | 70 | 9 |  |  |  |  |  |
| rs6588147 | 65935494 | AA | 668 | 106 | 0.99 | 0.70 | 1.41 | 0.964 | 0.965 |
|  |  | AG+GG | 405 | 59 |  |  |  |  |  |
| rs6657868 | 65913707 | GG | 345 | 46 | 1.18 | 0.81 | 1.73 | 0.391 | 0.385 |
|  |  | GA+AA | 728 | 118 |  |  |  |  |  |
| rs6669354 | 65925349 | TT | 775 | 125 | 0.77 | 0.52 | 1.15 | 0.203 | 0.205 |
|  |  | TG+GG | 298 | 40 |  |  |  |  |  |
| rs6673591 | 66048389 | AA | 279 | 46 | 0.92 | 0.62 | 1.35 | 0.660 | 0.663 |
|  |  | AG+GG | 792 | 119 |  |  |  |  |  |
| rs6678033 | 66077624 | GG | 275 | 37 | 1.18 | 0.78 | 1.77 | 0.428 | 0.431 |
|  |  | GA+AA | 785 | 125 |  |  |  |  |  |
| rs6688776 | 66068177 | AA | 287 | 48 | 0.94 | 0.64 | 1.38 | 0.747 | 0.746 |
|  |  | AG+GG | 786 | 118 |  |  |  |  |  |
| rs6694528 | 65963016 | CC | 483 | 80 | 0.85 | 0.60 | 1.20 | 0.345 | 0.345 |
|  |  | CT+TT | 590 | 85 |  |  |  |  |  |
| rs72921463 | 65936181 | GG | 784 | 123 | 0.87 | 0.59 | 1.29 | 0.479 | 0.484 |
|  |  | GA+AA | 289 | 42 |  |  |  |  |  |
| rs72925327 | 66100322 | GG | 874 | 137 | 0.74 | 0.46 | 1.19 | 0.219 | 0.218 |
|  |  | GT+TT | 198 | 28 |  |  |  |  |  |
| rs74081930 | 66077617 | TT | 963 | 151 | 0.75 | 0.40 | 1.39 | 0.359 | 0.352 |
|  |  | TC+CC | 109 | 14 |  |  |  |  |  |
| rs74084010 | 65998572 | GG | 963 | 147 | 0.99 | 0.56 | 1.73 | 0.967 | 0.988 |
|  |  | GA+AA | 110 | 18 |  |  |  |  |  |
| rs74733149 | 66075586 | AA | 1011 | 158 | 0.78 | 0.33 | 1.85 | 0.570 | 0.565 |
|  |  | AG+GG | 57 | 6 |  |  |  |  |  |
| rs7513047 | 65950340 | GG | 643 | 97 | 1.11 | 0.79 | 1.58 | 0.547 | 0.547 |
|  |  | GA+AA | 428 | 68 |  |  |  |  |  |
| rs75169973 | 65936244 | TT | 543 | 85 | 0.94 | 0.67 | 1.33 | 0.740 | 0.739 |
|  |  | TC+CC | 528 | 80 |  |  |  |  |  |
| rs7534511 | 65895130 | GG | 642 | 101 | 1.03 | 0.73 | 1.46 | 0.876 | 0.877 |
|  |  | GA+AA | 431 | 64 |  |  |  |  |  |
| rs75465244 | 65894940 | AA | 815 | 120 | 1.15 | 0.78 | 1.69 | 0.475 | 0.476 |
|  |  | AG+GG | 258 | 45 |  |  |  |  |  |
| rs7555955 | 65937479 | GG | 665 | 101 | 1.12 | 0.79 | 1.59 | 0.515 | 0.513 |
|  |  | GA+AA | 408 | 64 |  |  |  |  |  |
| rs75730881 | 65888973 | GG | 1025 | 160 | 0.70 | 0.27 | 1.81 | 0.465 | 0.389 |
|  |  | GA+AA | 48 | 5 |  |  |  |  |  |
| rs7602 | 65897951 | GG | 467 | 75 | 0.85 | 0.61 | 1.21 | 0.371 | 0.372 |
|  |  | GA+AA | 604 | 90 |  |  |  |  |  |
| rs77248581 | 66074891 | AA | 972 | 156 | 0.55 | 0.27 | 1.13 | 0.102 | 0.096 |
|  |  | AG+GG | 101 | 9 |  |  |  |  |  |
| rs77605029 | 65934080 | TT | 1008 | 157 | 0.81 | 0.36 | 1.79 | 0.600 | 0.642 |
|  |  | TG+GG | 62 | 8 |  |  |  |  |  |
| rs77848204 | 65894813 | GG | 840 | 122 | 1.22 | 0.83 | 1.81 | 0.316 | 0.319 |
|  |  | GA+AA | 233 | 43 |  |  |  |  |  |
| rs77980027 | 65922084 | TT | 923 | 150 | 0.57 | 0.32 | 1.02 | 0.059 | 0.059 |
|  |  | TC+C | 148 | 15 |  |  |  |  |  |
| rs78005150 | 65891384 | AA | 976 | 158 | 0.41 | 0.17 | 0.95 | **0.038** | **0.039** |
|  |  | AG+GG | 97 | 7 |  |  |  |  |  |
| rs7883 | 65897869 | GG | 649 | 97 | 0.94 | 0.66 | 1.33 | 0.709 | 0.711 |
|  |  | GA+AA | 424 | 68 |  |  |  |  |  |
| rs79353784 | 65979000 | GG | 1050 | 157 | 2.84 | 1.19 | 6.75 | **0.019** | **0.016** |
|  |  | GA+AA | 22 | 8 |  |  |  |  |  |
| rs79395241 | 65897838 | TT | 1047 | 161 | 1.20 | 0.41 | 3.53 | 0.740 | 0.778 |
|  |  | TC+CC | 24 | 4 |  |  |  |  |  |
| rs79896327 | 66097938 | CC | 1029 | 159 | 1.00 | 0.41 | 2.41 | 0.994 | 0.977 |
|  |  | CT+TT | 42 | 6 |  |  |  |  |  |
| rs8179183 | 66075952 | GG | 656 | 97 | 1.06 | 0.74 | 1.52 | 0.756 | 0.752 |
|  |  | GC+CC | 391 | 63 |  |  |  |  |  |
| rs9436297 | 65888854 | TT | 809 | 122 | 1.14 | 0.77 | 1.68 | 0.514 | 0.520 |
|  |  | TC+CC | 261 | 43 |  |  |  |  |  |
| rs9436299 | 65892888 | AA | 655 | 102 | 1.08 | 0.76 | 1.54 | 0.665 | 0.668 |
|  |  | AC+CC | 413 | 62 |  |  |  |  |  |
| rs9436300 | 65895264 | GG | 642 | 101 | 1.03 | 0.73 | 1.46 | 0.876 | 0.877 |
|  |  | GA+AA | 431 | 64 |  |  |  |  |  |
| rs9436301 | 65895927 | TT | 449 | 74 | 0.82 | 0.58 | 1.15 | 0.248 | 0.249 |
|  |  | TC+CC | 623 | 91 |  |  |  |  |  |
| rs9436302 | 65896561 | GG | 469 | 75 | 0.86 | 0.61 | 1.21 | 0.389 | 0.390 |
|  |  | GA+AA | 604 | 90 |  |  |  |  |  |
| rs9436738 | 65888560 | GG | 832 | 123 | 1.23 | 0.83 | 1.82 | 0.304 | 0.306 |
|  |  | GA+AA | 241 | 42 |  |  |  |  |  |
| rs9436742 | 65898331 | CC | 981 | 155 | 0.64 | 0.30 | 1.33 | 0.232 | 0.215 |
|  |  | CT+TT | 91 | 10 |  |  |  |  |  |
| rs9436746 | 65908473 | AA | 290 | 51 | 0.82 | 0.57 | 1.19 | 0.304 | 0.305 |
|  |  | AC+CC | 782 | 114 |  |  |  |  |  |
| rs9436748 | 65911672 | GG | 575 | 88 | 1.04 | 0.74 | 1.47 | 0.811 | 0.811 |
|  |  | GT+TT | 497 | 77 |  |  |  |  |  |
| rs9660088 | 66004606 | CC | 930 | 145 | 0.86 | 0.51 | 1.46 | 0.584 | 0.590 |
|  |  | CT+TT | 143 | 20 |  |  |  |  |  |
| rs970467 | 65906762 | GG | 699 | 115 | 0.74 | 0.51 | 1.08 | 0.116 | 0.115 |
|  |  | GA+AA | 373 | 50 |  |  |  |  |  |
| rs970468 | 65906490 | AA | 320 | 44 | 1.14 | 0.78 | 1.68 | 0.506 | 0.506 |
|  |  | AC+CC | 571 | 121 |  |  |  |  |  |

p value: adjusted by sex, age, energy, PC1, PC2, PC3

p*= Test permutacional (50 thousand permutations), dominant model
