# Supplementary material for: Food Consumption as a Modifier of the Association between LEPR Gene Variants and Excess Body Weight in Children and Adolescents: A Study of the SCAALA Cohort
Source: Nutrients. 2018 Aug 18;10(8):1117. doi: 10.3390/nu10081117 (PMC6116060; doi:10.3390/nu10081117)
Supplement: Supplementary file 1 [file nutrients-10-01117-s001.zip › Supplementary table 2.docx]

**Supplementary table 2:** Association between *LEPR* gene variants and excess weight according to dietary intake. Salvador, Bahia, Brazil, 2005–2006.

| SNVs | | OR | 95% CI | P_interaction_ |
| --- | --- | --- | --- | --- |
| Protein (% calorie) | | | | |
| rs1177681 | < Median | 1.08 | 0.63 - 1.87 | 0.282 |
|  | ≥ Median | **1.64** | **1.03 - 2.61** |  |
| rs1137100 | < Median | 1.06 | 0.60 - 1.87 | 0.348 |
|  | ≥ Median | 1.54 | 0.96 - 2.46 |  |
| rs1137101 | < Median | 0.62 | 0.36 - 1.10 | 0.046 |
|  | ≥ Median | 1.32 | 0.76 - 2.30 |  |
| rs8179183 | < Median | 1.14 | 0.65 – 2.01 | 0.939 |
|  | ≥ Median | 1.06 | 0.64 - 1.74 |  |
| rs78005150 | < Median | 0.30 | 0.07 - 1.30 | 0.716 |
|  | ≥ Median | 0.46 | 0.16 - 1.33 |  |
| rs116239759 | < Median | 1.06 | 0.31- 3.70 | 0.073 |
|  | ≥ Median | **4.53** | **1.74 - 11.76** |  |
|  |  |  |  |  |
| rs202069668 | < Median | 1.39 | 0.82 - 2.45 | 0.891 |
|  | ≥ Median | 1.47 | 0.93 – 2.34 |  |
|  |  |  |  |  |
| rs79353784 | < Median | 1.09 | 0.13 – 9.04 | 0.085 |
|  | ≥ Median | **4.33** | **1.59- 11.80** |  |
| rs115650230 | < Median | 1.55 | 0.51 - 4.76 | 0.419 |
|  | ≥ Median | **3.07** | **1.19 - 7.95** |  |
| Fat (% calorie) | | | | |
| rs1177681 | < Median | 1.02  1.81 | 0.59 - 1.77 | 0.112 |
|  | ≥ Median |  | 1.14 - 2.89 |  |
| rs1137100 | < Median | 1.09 | 0.62 - 1.92 | 0.304 |
|  | ≥ Median | **1.57** | **0.98 - 2.51** |  |
| rs1137101 | < Median | 0.90 | 0.50 – 1.63 | 0.841 |
|  | ≥ Median | 0.97 | 0.58 - 1.62 |  |
| rs8179183 | < Median | 0.88 | 0.49 - 1.55 | 0.237 |
|  | ≥ Median | 1.33 | 0.81 - 2.18 |  |
| rs78005150 | < Median | 0.15 | 0.02 - 1.17 | 0.246 |
|  | ≥ Median | 0.56 | 0.22 - 1.48 |  |
| rs116239759 | < Median | 1.11 | 0.31 - 3.95 | 0.148 |
|  | ≥ Median | 3.61 | 1.45 - 9.00 |  |
| rs202069668 |  |  |  | 0.311 |
|  | < Median | 1.21 | 0.72 - 2.05 |  |
|  | ≥ Median | 1.72 | 1.08 - 2.74 |  |
|  |  |  |  |  |
| rs79353784 | < Median | 5.24 | 1.80 - 15.31 | 0.273 |
|  | ≥ Median | 1.58 | 0.33 - 7.62 |  |
| rs115650230 | < Median | 1.00 | 0.28 - 3.52 | 0.081 |
|  | ≥ Median | 4.06 | 1.60 - 10.35 |  |
| Carbohydrate (% calorie) | | | | |
| rs1177681 | < Median | **1.69** | **1.05 - 2.73** | 0.270 |
|  | ≥ Median | 1.11 | 0.66 - 1.88 |  |
| rs1137100 | < Median | 1.52 | 0.94 - 2.48 | 0.440 |
|  | ≥ Median | 1.14 | 0.67 - 1.96 |  |
| rs1137101 | < Median | 1.05 | 0.62 - 1.78 | 0.550 |
|  | ≥ Median | 0.83 | 0.47 - 1.45 |  |
| rs8179183 | < Median | ------ | ------- | 0.660 |
|  | ≥ Median | ----- | ------- |  |
| rs78005150 | < Median | 0.62 | 0.24 - 1.62 | 0.165 |
|  | ≥ Median | 0.13 | 0.02 - 1.05 |  |
| rs116239759 | < Median | **3.50** | **1.43 - 8.58** | 0.143 |
|  | ≥ Median | 1.08 | 0.30 - 3.91 |  |
| rs202069668 |  |  |  | 0.311 |
|  | < Median | **1.88** | **1.16 - 3.05** |  |
|  | ≥ Median | 1.19 | 0. 67 - 1.85 |  |
|  |  |  |  |  |
| rs79353784 | < Median | 2.95 | 0.74 - 11.74 | 0.962 |
|  | ≥ Median | **3.55** | **1.17 - 10.76** |  |
| rs115650230 | < Median | **3.94** | **1.58 - 9.82** | 0.078 |
|  | ≥ Median | 0.96 | 0.27 - 3.44 |  |
| PUFA:SAT ratio | | | | |
| rs1177681 | < Median | **1.97** | **1.21 - 3.20** | **0.050** |
|  | ≥ Median | 0.98 | 0.58 - 1.64 |  |
| rs1137100 | < Median | 1.92 | 1.18 - 3.14 | **0.037** |
|  | ≥ Median | 0.90 | 0.02 - 1.55 |  |
| rs1137101 | < Median | 1.18 | 0.06 - 2.11 | 0.285 |
|  | ≥ Median | 0.77 | 0.45 - 1.28 |  |
| rs8179183 | < Median | 0.88 | 0.52 – 1.50 | 0.336 |
|  | ≥ Median | ----- | ------- |  |
| rs78005150 | < Median | 0.44 | 0.13 - 1.46 | 0.877 |
|  | ≥ Median | 0.37 | 0.11 – 1.23 |  |
| rs116239759 | < Median | 1.78 | 0.70 - 4.62 | 0.469 |
|  | ≥ Median | 3.35 | 1.12 – 10.06 |  |
| rs202069668 | < Median | 1.91 | 1.16 - 3.13 | 0.149 |
|  | ≥ Median | 1.14 | 0.70 - 1.87 |  |
| rs79353784 | < Median | 3.79 | 1.10 - 13.06 | 0.562 |
|  | ≥ Median | 2.80 | 0.84 - 9.39 |  |
| rs115650230 | < Median | 1.89 | 0.73 - 4.89 | 0.637 |
|  | ≥ Median | 2.80 | 0.95 - 8.29 |  |

----- non-converged analyzes

*****p interaction: likelihood ratio test adjusted for sex, age, energy, PC1, PC2, PC3
