# Supplementary material for: Food Consumption as a Modifier of the Association between LEPR Gene Variants and Excess Body Weight in Children and Adolescents: A Study of the SCAALA Cohort
Source: Nutrients. 2018 Aug 18;10(8):1117. doi: 10.3390/nu10081117 (PMC6116060; doi:10.3390/nu10081117)
Supplement: Supplementary file 1 [file nutrients-10-01117-s001.zip › Supplementary table 3.docx]

**Supplementary table 3:** Association between LEPR gene variants and excess weight according to food group. Salvador, Bahia, Brazil, 2005–2006.

| SNVs | | OR | 95% CI | P _interaction_ |
| --- | --- | --- | --- | --- |
| Milk and dairy products | | | | |
| rs1177681 | < Median | 1.04 | 0.60 - 1.80 | 0.119 |
|  | ≥ Median | **1.85** | **1.16 - 2.94** |  |
| rs1137100 | < Median | 1.29 | 0.74 - 2.25 | 0.777 |
|  | ≥ Median | 1.45 | 0.90 - 2.33 |  |
| rs1137101 | < Median | ------ | ------- | 0.380 |
|  | ≥ Median | 0.82 | 0.51 - 1.35 |  |
| rs8179183 | < Median | 1.10 | 0.61 - 1.95 | 0.922 |
|  | ≥ Median | 1.07 | 0.66 - 1.76 |  |
| rs78005150 | < Median | 0.49 | 0.15 - 1.63 | 0.743 |
|  | ≥ Median | 0.36 | 0.11 - 1.24 |  |
| rs116239759 | < Median | 1.15 | 0.33 - 3.96 | 0.073 |
|  | ≥ Median | **4.60** | **1.73 - 12.25** |  |
| rs202069668 | < Median | ------ | ----------- | 0.826 |
|  | ≥ Median | 1.45 | 0.92 - 2.28 |  |
| rs79353784 | < Median | ------- | ---------- | 0.599 |
|  | ≥ Median | **3.63** | **1.15 - 11.49** |  |
| rs115650230 | < Median | 1.10 | 0.32 - 3.79 | 0.077 |
|  | ≥ Median | 4.39 | 1.66 - 11.62 |  |
| Processed meats group | | | | |
| rs1177681 | < Median | 1.28 | 0.79 - 2.09 | 0.584 |
|  | ≥ Median | 1.59 | 0.95 - 2.64 |  |
| rs1137100 | < Median | 1.22 | 0.74 - 2.03 | 0.605 |
|  | ≥ Median | 1.48 | 0.88 - 2.49 |  |
| rs1137101 | < Median | 0.79 | 0.47 - 1.33 | 0.460 |
|  | ≥ Median | 1.09 | 0.61 - 1.94 |  |
| rs8179183 | < Median | 1.02 | 0.61 - 1.71 | 0.839 |
|  | ≥ Median | 1.10 | 0.64 - 1.89 |  |
| rs78005150 | < Median | 0.38 | 0.11 - 1.26 | 0.903 |
|  | ≥ Median | 0.42 | 0.13 - 1.42 |  |
| rs116239759 | < Median | 1.91 | 0.75 - 4.88 | 0.476 |
|  | ≥ Median | 3.10 | 0.99 - 9.70 |  |
| rs202069668 | < Median | 1.55 | 0.95 - 2.50 | 0.852 |
|  | ≥ Median | 1.41 | 0.86 - 2.33 |  |
| rs79353784 | < Median | 2.77 | 0.70 - 10.95 | 0.640 |
|  | ≥ Median | **3.30** | **1.08 - 10.09** |  |
| rs115650230 | < Median | 1.89 | 0.74 - 4.85 | 0.628 |
|  | ≥ Median | 2.74 | 0.90 - 8.38 |  |
| Fried foods group | | | | |
| rs1177681 | < Median | 1.50 | 0.88 - 2.78 | 0.730 |
|  | ≥ Median | 1.32 | 0.83 - 2.10 |  |
| rs1137100 | < Median | 1.43 | 0.83 - 2.47 | 0.730 |
|  | ≥ Median | 1.25 | 0.78 - 2.02 |  |
| rs1137101 | < Median | 1.23 | 0.65 - 2.33 | 1.000 |
|  | ≥ Median | 0.79 | 0.48 - 1.29 |  |
| rs8179183 | < Median | -------- | ------------ | 0.363 |
|  | ≥ Median | -------- | ------------ |  |
| rs78005150 | < Median | 0.17 | 0.02 - 1.25 | 0.236 |
|  | ≥ Median | 0.56 | 0.22 - 1.44 |  |
| rs116239759 | < Median | 2.01 | 0.71 - 5.69 | 0.590 |
|  | ≥ Median | **2.75** | **1.02 - 7.42** |  |
| rs202069668 | < Median | ----- | -------- | 0.624 |
|  | ≥ Median | ------ | --------- |  |
| rs79353784 | < Median | ----- | -------- | 0.816 |
|  | ≥ Median | ------ | --------- |  |
| rs115650230 | < Median | ----- | -------- | 0.737 |
|  | ≥ Median | ------ | --------- |  |
| Chicken group | | | | |
| rs1177681 | < Median | 1.48 | 0.95 - 2.33 | 0.734 |
|  | ≥ Median | 1.25 | 0.72 - 2.19 |  |
| rs1137100 | < Median | 1.27 | 0.80 - 2.01 | 0.655 |
|  | ≥ Median | 1.44 | 0.81 - 2.55 |  |
| rs1137101 | < Median | 1.14 | 0.67 - 1.94 | 0.327 |
|  | ≥ Median | 0.78 | 0.44 - 1.39 |  |
| rs8179183 | < Median | 1.01 | 0.63 - 1.63 | 0.566 |
|  | ≥ Median | 1.25 | 0.69 - 2.24 |  |
| rs78005150 | < Median | 0.49 | 0.17 - 1.38 | 0.530 |
|  | ≥ Median | 0.26 | 0.06 - 1.15 |  |
| rs116239759 | < Median | **2.70** | **1.03 - 7.08** | 0.611 |
|  | ≥ Median | 1.88 | 0.66 - 5.41 |  |
| rs202069668 | < Median | 1.35 | 0.87 - 2.12 | 0.529 |
|  | ≥ Median | 1.65 | 0.96 - 2.86 |  |
| rs79353784 | < Median | **3.62** | **1.39 - 9.45** | 0.376 |
|  | ≥ Median | 1.42 | 0.16 - 12.69 |  |
| rs115650230 | < Median | 2.34 | 0.91 - 6.07 | 0.857 |
|  | ≥ Median | 2.09 | 0.72 - 6.08 |  |
| Sweets group | | | | |
| rs1177681 | < Median | 1.31 | 0.80 - 2.14 | 0.744 |
|  | ≥ Median | 1.45 | 0.88 - 2.40 |  |
| rs1137100 | < Median | 1.22 | 0.73 - 2.02 | 0.588 |
|  | ≥ Median | 1.46 | 0.87 - 2.43 |  |
| rs1137101 | < Median | 0.92 | 0.54 - 1.58 | 0.922 |
|  | ≥ Median | 1.00 | 0.57 - 1.76 |  |
| rs8179183 | < Median | 1.38 | 0.83 - 2.30 | 0.188 |
|  | ≥ Median | 0.87 | 0.51 - 1.51 |  |
| rs78005150 | < Median | 0.48 | 0.14 - 1.59 | 0.788 |
|  | ≥ Median | 0.32 | 0.09 - 1.10 |  |
| rs116239759 | < Median | 2.15 | 0.81 - 5.67 | 0.931 |
|  | ≥ Median | 2.23 | 0.77 - 6.44 |  |
| rs202069668 | < Median | 1.20 | 0.74 - 1.93 | 0.198 |
|  | ≥ Median | **1.81** | **1.09 - 3.01** |  |
| rs79353784 | < Median | 2.89 | 0.92 - 9.10 | 0.893 |
|  | ≥ Median | 2.58 | 0.67 - 9.98 |  |
| rs115650230 | < Median | 2.45 | 0.92 - 6.54 | 0.740 |
|  | ≥ Median | 1.94 | 0.68 - 5.53 |  |
| Legumes group | | | | |
| rs1177681 | < Median | 1.21 | 0.66 - 2.24 | 0.590 |
|  | ≥ Median | 1.50 | 0.98 - 2.30 |  |
| rs1137100 | < Median | 1.26 | 0.66 - 2.40 | 0.777 |
|  | ≥ Median | 1.38 | 0.89 - 2.13 |  |
| rs1137101 | < Median | 0.75 | 0.40 - 1.43 | 0.777 |
|  | ≥ Median | 1.07 | 0.66 - 1.73 |  |
| rs8179183 | < Median | 0.96 | 0.51 - 1.81 | 0.594 |
|  | ≥ Median | 1.20 | 0.76 - 1.90 |  |
| rs78005150 | < Median | 0.74 | 0.27 - 2.06 | 0.069 |
|  | ≥ Median | 0.13 | 0.02 - 0.93 |  |
| rs116239759 | < Median | 2.03 | 0.61 - 6.80 | 0.856 |
|  | ≥ Median | **2.45** | **1.00 - 6.01** |  |
| rs202069668 | < Median | 1.29 | 0.71 - 2.34 | 0.487 |
|  | ≥ Median | **1.63** | **1.07 - 2.50** |  |
| rs79353784 | < Median | 2.84 | 0.52 - 15.43 | 0.991 |
|  | ≥ Median | 2.84 | 1.03 - 7.82 |  |
| rs115650230 | < Median | 1.64 | 0.51 - 5.31 | 0.520 |
|  | ≥ Median | **2.76** | **1.11 - 6.88** |  |
| Refined cereals group | | | | |
| rs1177681 | < Median | 0.75 | 0.44 - 1.25 | **0.000** |
|  | ≥ Median | **2.74** | **1.64 - 4.57** |  |
| rs1137100 | < Median | 0.84 | 0.50 - 1.43 | **0.011** |
|  | ≥ Median | **2.17** | **1.30 - 3.62** |  |
| rs1137101 | < Median | 0.78 | 0.47 - 1.32 | 0.281 |
|  | ≥ Median | 1.21 | 0.67 - 2.18 |  |
| rs8179183 | < Median | **1.75** | **1.75 - 2.90** | **0.005** |
|  | ≥ Median | 0.63 | 0.35 - 1.12 |  |
| rs78005150 | < Median | 0.18 | 0.02 - 1.34 | 0.265 |
|  | ≥ Median | 0.55 | 0.01 - 0.09 |  |
| rs116239759 | < Median | 1.26 | 0.41 - 3.82 | 0.121 |
|  | ≥ Median | **3.98** | **1.53 - 10.37** |  |
| rs202069668 | < Median | 0.91 | 0.57 - 1.47 | **0.003** |
|  | ≥ Median | **2.66** | **1.56 - 4.54** |  |
| rs79353784 | < Median | 0.87 | 0.17 - 4.52 | **0.042** |
|  | ≥ Median | **6.42** | **2.06 - 20.07** |  |
| rs115650230 | < Median | **2.06** | **0.72 - 5.86** | 0.840 |
|  | ≥ Median | 2.41 | 0.91 - 6.35 |  |
| Egg group | | | | |
| rs1177681 | < Median | **1.53** | **1.04 - 2.24** | 0.260 |
|  | ≥ Median | 0.83 | 0.35 - 2.00 |  |
| rs1137100 | < Median | 1.38 | 0.93 - 2.05 | 0.642 |
|  | ≥ Median | 1.00 | 0.41 - 2.41 |  |
| rs1137101 | < Median | 1.08 | 0.69 - 1.67 | 0.122 |
|  | ≥ Median | 0.48 | 0.20 - 1.15 |  |
| rs8179183 | < Median | 1.02 | 0.67 - 1.54 | 0.356 |
|  | ≥ Median | 1.77 | 0.74 - 4.22 |  |
| rs78005150 | < Median | 0.48 | 0.20 - 1.14 | 0.356 |
|  | ≥ Median | ------ | ----- |  |
| rs116239759 | < Median | **2.25** | **1.03 – 4.94** | 0.900 |
|  | ≥ Median | 1.91 | 0.34 - 10.85 |  |
| rs202069668 | < Median | **1.68** | **1.14 - 2.46** | 0.134 |
|  | ≥ Median | 0.73 | 0.31 - 1.74 |  |
| rs79353784 | < Median | 1.93 | 0.67 - 5.55 | 0.105 |
|  | ≥ Median | **8.35** | **1.28 - 54.41** |  |
| rs115650230 | < Median | 2.07 | 0.95 - 4.51 | 0.689 |
|  | ≥ Median | 2.59 | 0.45 - 14.93 |  |
| Read meat group | | | | |
| rs1177681 | < Median | 1.32 | 0.78 - 2.24 | 0.695 |
|  | ≥ Median | 1.52 | 0.94 - 2.44 |  |
| rs1137100 | < Median | 1.16 | 0.67 - 2.00 | 0.442 |
|  | ≥ Median | 1.54 | 0.95 - 2.49 |  |
| rs1137101 | < Median | 0.88 | 0.50 - 1.57 | 0.672 |
|  | ≥ Median | 0.99 | 0.59 - 1.68 |  |
| rs8179183 | < Median | 0.95 | 0.53 - 1.71 | 0.676 |
|  | ≥ Median | 1.14 | 0.71 - 1.86 |  |
| rs78005150 | < Median | 0.46 | 0.14 - 1.51 | 0.773 |
|  | ≥ Median | 0.35 | 0.11 - 1.20 |  |
| rs116239759 | < Median | **2.50** | **1.02 - 6.15** | 0.734 |
|  | ≥ Median | 2.12 | 0.66 - 6.85 |  |
| rs202069668 | < Median | 1.36 | 0.81 - 2.29 | 0. 612 |
|  | ≥ Median | 1.62 | 1.02 - 2.58 |  |
| rs79353784 | < Median | 1.29 | 0.24 - 6.84 | 0.293 |
|  | ≥ Median | 4.03 | 1.39 - 11.73 |  |
| rs115650230 | < Median | **3.01** | **1.28 - 7.11** | 0.294 |
|  | ≥ Median | 1.34 | 0.36 - 4.92 |  |
| Soft drinks/artificial juices group | | | | |
| rs1177681 | < Median | 1.23 | 0.77 - 1.96 | 0.394 |
|  | ≥ Median | 1.67 | 0.98 - 2.85 |  |
| rs1137100 | < Median | 0.91 | 0.55 - 1.50 | **0.019** |
|  | ≥ Median | **2.15** | **1.26 - 3.68** |  |
| rs1137101 | < Median | 0.96 | 0.57 - 1.62 | 0.732 |
|  | ≥ Median | 0.89 | 0.50 - 1.60 |  |
| rs8179183 | < Median | 1.24 | 0.77 - 2.03 | 0.412 |
|  | ≥ Median | 0.92 | 0.51 - 1.66 |  |
| rs78005150 | < Median | 0.58 | 0.20 - 1.67 | 0.359 |
|  | ≥ Median | 0.23 | 0.05 - 1.00 |  |
| rs116239759 | < Median | 2.62 | 1.05 - 6.49 | 0.767 |
|  | ≥ Median | 1.93 | 0.61 - 6.11 |  |
| rs202069668 | < Median | 1.11 | 0.70 - 1.75 | 0.061 |
|  | ≥ Median | **2.10** | **1.22 - 3.61** |  |
| rs79353784 | < Median | 3.08 | 1.00 - 9.49 | 0.990 |
|  | ≥ Median | 2.97 | 0.73 - 12.07 |  |
| rs115650230 | < Median | **2.55** | **1.02 - 6.35** | 0.704 |
|  | ≥ Median | 1.93 | 0.61 - 6.07 |  |
| Margarine group | | | | |
| rs1177681 | < Median | 1.18 | 0.66 - 2.11 | 0.485 |
|  | ≥ Median | **1.56** | **1.00 - 2.42** |  |
| rs1137100 | < Median | 1.36 | 0.76 - 2.45 | 0.999 |
|  | ≥ Median | 1.34 | 0.85 - 2.12 |  |
| rs1137101 | < Median | 1.38 | 0.71 - 2.67 | 0.151 |
|  | ≥ Median | 0.76 | 0.47 - 1.22 |  |
| rs8179183 | < Median | 1.01 | 0.56 - 1.83 | 0.840 |
|  | ≥ Median | 1.15 | 0.72 - 1.84 |  |
| rs78005150 | < Median | 0.32 | 0.07 - 1.39 | 0.662 |
|  | ≥ Median | 0.46 | 0.16 - 1.32 |  |
| rs116239759 | < Median | 2.16 | 0.66 - 7.06 | 0.879 |
|  | ≥ Median | 2.33 | 0.96 - 5.65 |  |
| rs202069668 | < Median | 1.20 | 0.69 - 2.09 | 0.376 |
|  | ≥ Median | **1.68** | **1.08 - 2.62** |  |
| rs79353784 | < Median | 2.99 | 0.85 - 10.54 | 0.897 |
|  | ≥ Median | 2.57 | 0.75 - 8.85 |  |
| rs115650230 | < Median | 2.54 | 0.73 - 8.79 | 0.667 |
|  | ≥ Median | 2.01 | 0.84 - 4.81 |  |

----- non-converged analyzes

*****P interaction: likelihood ratio test adjusted for sex. age. energy. PC1. PC2. PC3
